# An Empirical and Subjective Model of Upper Extremity Fatigue Under Hypogravity

**DOI:** 10.3389/fphys.2022.832214

**Published:** 2022-02-16

**Authors:** Tatiana Volkova, Claude Nicollier, Volker Gass

**Affiliations:** Space Innovation, École Polytechnique Fédérale de Lausanne, Lausanne, Switzerland

**Keywords:** reduced gravity, partial gravity, workplace, fatigue models, numerical simulation, sitting posture, biomechanics, underwater

## Abstract

In the context of extra-terrestrial missions, the effects of hypogravity (0 < G < 1) on the human body can reduce the well-being of the crew, cause musculoskeletal problems and affect their ability to perform tasks, especially during long-term missions. To date, studies of the effects of hypogravity on human movement are limited to experiments on the lower limbs. Here, we extend the knowledge base to the upper limbs, by conducting experiments to evaluate the effect of hypogravity on upper limb physical fatigue and mental workload in participants. Our hypothesis was that hypogravity would both increase participant productivity, by reducing overall physical fatigue expressed in Endurance Time, and reduce mental workload. Task Intensity-Endurance time curves are developed especially in seated positions, while performing static, dynamic, repetitive tasks. This experiment involved 32 healthy participants without chronic problems of the musculoskeletal system aged 33.59 ± 8.16 years. Using the collected data, fatigue models were constructed for tasks of varying Intensity. In addition, all participants completed the NASA – Task Load Index subjective mental workload assessment, which revealed the level of subjective workload when executing different tasks. We found two trends in the empirical fatigue models associated with the difference between the strength capabilities of males and females. The first is a significant positive (*p* = 0.002) relation between Endurance time and gravity level (⅙ G Moon, ⅓ G Mars, 1G) with negative coefficient for males and females for a static task. And there is marginal relation (*p* < 0.1) between overall mental workload and gravity level with a positive coefficient for males and females for the same task. The same trend was observed for dynamic and repetitive tasks. We concluded that the Task Intensity-Endurance Time model, adapted to hypogravity in combination with subjective mental assessment, is useful to human fatigue investigation. The combination of these methods used for ergonomic analysis and digital human modeling, could improve worker productivity. Finally, this study may help prepare astronauts for long-term missions on the Moon and Mars and improve our understanding of how we can prevent musculoskeletal disorders caused by hazardous manual handling under such extreme environments.

## 1. Introduction

As we return to the Moon and aim for Mars, the effect of hypogravity (HG) on the human body will play a crucial role (Riley et al., 1987; D'Aunno et al., 2003; Horneck et al., 2003; Orwoll et al., 2013; Widjaja et al., 2015; Clément, 2017; Reynolds, 2019; De Martino et al., 2020; Swanenburg et al., 2020) whether running, walking, or sitting. To the best of the authors' knowledge, research on the effects of hypogravity on human movement has only focused on the lower limbs (Hewes and Spady, 1964; Rajulu and Klute, 1992; Newman and Alexander, 1993; Sylos-Labini et al., 2014; Richter et al., 2017; Kang et al., 2019; Weber et al., 2019)with little information about the upper extremities under HG and in particular, during manual handling operations.

In spite of significant automation in industrial environments, muscle strength remains an integral part of many working operations (De Looze et al., 2016). To a large extent, such work involves manual handling of equipment and maintenance work. Manual handling of heavy objects can cause high loads on the musculoskeletal system, potentially leading to accidents (Edlich et al., 2005; Clarke, 2020), lost time and additional costs. There were registered (15.8%) injuries during heavy lifting and 16.9% of accidents associated with lumbar and back injuries (Spengler et al., 1986; Clarke, 2020; Wizner et al., 2021) on Earth. At the same time, such parameters as the type of task, size, shape and weight of the manual tool play an important role in the impact on the spine and upper limbs. According to Hernandez et al. (2019) astronaut injuries associated with spacesuit wearing have been reported. For example, potential risk for shoulder injuries in space can be overhead tasks.

In the context of extra-terrestrial missions under HG, musculoskeletal, as well as cardiovascular, vestibular systems will be affected because of gravity changes (Morey-Holton, 2003). According to Axpe et al. (2020) body mineral density (BMD) is one of the most important components used by ISS crewmembers to monitor bone health. Based on the collected data on board the International Space Station (ISS), Axpe et al. (2020) predicted with non-linear exponential model during 6 months of flight to Mars, and during long stays on Mars and on the Moon, astronauts may lose approximately 32.4–36.8% of the bone mineral density in the femoral neck. If such measurements are completed after 6 months of experimentation, similar results are expected. According to Morey-Holton (2003), bones and muscles are lost only in the lower limbs and upper limbs including the back. This suggests that changes in the musculoskeletal system are local.

Upper limbs have not been extensively researched. The first experiments related to the study of upper limb fatigue in weightlessness were conducted on parabolic flight (Bock, 1996) and on MIR (Gallasch et al., 1996). Upper limb experimentation is gaining in importance as part of missions to the ISS (Pastacaldi et al., 2004). This is due to a change in motor control programs in microgravity conditions, affectings not only the biomechanics of the astronauts, but in general their entire psychophysical state. Finally, another study (Nagatomo et al., 2014) based on parabolic flight study, simulating different levels of gravity (0G - 1.5G) compared the blood flow of the upper and lower extremities of seated participants, finding that blood flow in the upper extremities had a normal levels for microgravity, at the same time blood flow in the lower extremities decreased for 0 G. However, long-duration effects of HG on upper limbs were not analyzed.

During long missions, manual handling and repair work will be performed. The associated physiological changes and possible risks and injuries in the workplace should thus be studied and considered. It is necessary to assess physical fatigue and perform manual control. To the best of our knowledge, no such studies have yet been conducted on the work of the upper limb in conditions of HG.

Handling capacities can be assessed by directly measuring muscle fatigue. Muscle fatigue is designated as a decrease in the capacity to maintain the required level of strength after continuous muscle engagement (Boyas and Guével, 2011). This is a phenomenon that depends on many factors, including the characteristics of the task being performed (Mehta and Parasuraman, 2014). If not enough to recover, muscle fatigue reduces the tissue's ability to withstand stress. This can lead to musculoskeletal disorders (Kumar, 2001). Productivity can also be affected by muscle fatigue, which can then reduce workers' potential to develop different ways of working to achieve production goals without without prejudice to their health (Durand et al., 2009).

To assess physical fatigue, many empirical models can be built. Such models are described by Intensity - Endurance time (ET) curves with exponential or power function (Rohmert, 1960; Monod and Scherrer, 1965; Huijgens, 1981; Rose et al., 2000; Garg et al., 2002; Imbeau et al., 2006; Frey Law and Avin, 2010; Ma et al., 2011) used to calculate maximum holding time for any particular task. These models differ by joint regions (shoulder, elbow, wrist), where maximum strength is exponential due to the amount of energy the body is able to transfer to the muscles. According to Seo et al. (2016), such models can't be used for dynamic tasks studies with out of order pauses. For this, dynamic fatigue models were developed. One of such models was described by Liu et al. (2002) as s set of dynamic equations considering effect of muscle fatigue and recovery. Then Xia and Law (2008) defined a muscle fatigue mathematical model for complex tasks. Model of Ma et al. (2009) can be applied for static and dynamic tasks, with respect to specific body parts. And it estimates muscle ability based on the history of muscle activity of different body parts without fatigue recovery consideration.

To measure physiological parameters for upper extremity physical fatigue investigation, various tools, including the dynamometer (Alizadehkhaiyat et al., 2007; Romero-Franco et al., 2019), electromyogram (Chany et al., 2007; Lalitharatne et al., 2012), electroencephalographic measure (Wang et al., 2021) and electrocardiogram (Redgrave et al., 2018) can be used. The following monitoring approaches can also be applied: posture sway (Davidson et al., 2004), joint kinematics (Riley and Bilodeau, 2002), perceived discomfort/fatigue (Balci and Aghazadeh, 2004).

More subjective assessment methods can also be implemented, particularly for mental workload study and workplace ergonomic risk factors. Examples of such methods, applicable to specific case studies concerning pilots or astronauts, are: Cooper-Harper Scale (Cooper and Harper, 1969), the Bedford Scale (Roscoe and Ellis, 1990), the Subjective Assessment Technique (SWAT) (Reid and Nygren, 1988) and the NASA Task Load Index (NASA-TLX) (Hart and Staveland, 1988), the Workload Profile (WP) (Tsang and Velazquez, 1996). According to one study (Rubio et al., 2004) NASA-TLX has the highest sensitivity, as well as strongest operator acceptance (Hill et al., 1992) compared to SWAT, WP. Also, as a result of the same study comparing these three tools, NASA-TLX's validity assessment gave a positive correlation coefficient between them. Furthermore, NASA-TLX shows a higher correlation with human performance than SWAT and WP. Rubio et al. (2004) suggested that the SWAT and NASA-TLX is credible for composite natural world tasks. Rubio et al. (2004) concluded that if the goal is to predict human performance when performing a specific task, NASA-TLX is suggested because of its high correlation with this parameter.

The combination of Task Intensity - ET estimation and NASA-TLX formed the basis of the approach presented in this study. We assumed that HG would increase participant productivity, by reducing physical fatigue, and also reduce the mental workload of participants compared to Earth's gravity. Such a model can be used to assess physiological limitations of a given workplace environment in HG as well as 1G. For HG in particular, maximum admissible weight and forces for various percentages of a population can be designed based on this data, reducing the number of cumulative trauma and disorders related to specific motions. Although there is critically little data collected about the time of real experiments, considering the simulation of motion under HG, digital human modeling (DHM) is now widely used for testing various manual scenarios without real participants.

## 2. Materials and Methods

### 2.1. Participants

A total of 32 volunteers participated in the study (18 males, 14 females). All participants were right-hand dominant and reported that they did not currently have health issues. Two sets of experiments were conducted in a 1G and then underwater. Underwater conditions were used because Archimedes force counteracts the action of gravity.

During the experiments in the water tank (Swissub, Vaud, Switzerland) all participants were seated with their heads above the water level. The capacity of the parallelepiped water tank, adapted for experiments, is 8.3 tons. The two sides of the water tank are made of glass and the participant can be observed from the outside. The temperature in the water was constant at 29 degrees. Ballasts were selected for the different body parts to provide the necessary level of buoyancy, equivalent to gravity on the Moon (G = 1.626 m/s ^2^) and Mars (G = 3.72076 m/s ^2^).

### 2.2. Statistical Methods

Data distribution was analyzed using Excel and statistical analysis package Stata 17 (StataCorp, California, US). We used empirical and a subjective model for physical fatigue of upper extremity fatigue and mental workload assessment with three levels of gravity, six task intensities, and four types of tasks [outstretched arm (S1), arm bent at the elbow (S2), dynamic (D) and repetitive (R)], with gender as an independent variable, and Endurance time (min), mental workload, hand and back-chest-leg muscle contraction as dependent variables. Statistical significance was defined at α = 0.05. The power model were was used to determine the Endurance Time—Task Intensity curves for all types of tasks. In accordance with these models R-squared values and power model coefficients were investigated. Then an assessment of the normality of distribution, *p*-values calculation, one tailed paired samples *t*-test were conducted. To investigate the level of significance of the dependence between ET (min) and WWL,% and muscle contraction from the gravity level (1G, ⅓ G and ⅙ G), as well as the character of this dependence we conducted statistical analysis by means of a multivariate regression with correlation coefficients, standard errors and *p*-values assessment.

Continuous variables are presented as means and standard deviations. Effect sizes were calculated using G*Power software version (3.1.9.7). Thresholds were defined as 0.2 (small effect), 0.5 (moderate effect), and 0.8 (large effect) (Cohen, 1992) between four experimental groups. With 100 participants split in four different groups, a *post-hoc* power analysis suggests a power of 0.88 for average sized group effects. The power analysis is presented in Supplementary Section 1.1. Descriptive statistics of anthropometric data of the participants are presented below in Table 1.

**Table 1:** Descriptive statistics for the main characteristics of the participant.

**Study variable**	**Total (*N* = 32)**		**Male (*N* = 18)**	**Female (*N* = 14)**	***p*-value**
	**Mean (SD)**	**Min/Max**	**Mean (SD)**	**Mean (SD)**	
Age (year)	33.59 (8.16)	25/55	34 (9.62)	33.07 (6.11)	0.742
Height (m)	1.75 (0.11)	1.54/1.95	1.83 (0.07)	1.66 (0.06)	<0.001
Body mass (kg)	71.22 (17.01)	43.8/114.10	82.92 (13.02)	56.19 (5.95)	<0.001
BMI (kg/m ^2^)	22.91 (3.78)	16.09/37.43	24.83 (3.77)	20.43 (1.93)	<0.001
Muscle mass (kg)	53.64 (12.51)	35.10/75.80	63.3 (7.38)	41.22 (2.60)	<0.001
Body fat (%)	19.67 (6.29)	8.50/36.40	17.77 (6.46)	22.11 (5.32)	0.046
Body fat (kg)	14.18 (6.75)	5.36/41.53	15.38 (8.23)	12.65 (3.93)	0.227
Body water (%)	54.74 (6.13)	30.30/66.00	56.89 (4.52)	51.98 (6.94)	0.032
Body water (kg)	38.99 (9.97)	17.85/55.28	46.74(4.94)	29.02 (3.86)	<0.001
Bone mass (kg)	2.87 (0.65)	2.00/4.00	3.37 (0.38)	2.23 (0.12)	<0.001
Upper arm (m)	0.34 (0.04)	0.25/0.40	0.35 (0.33)	0.32 (0.03)	0.007
Forearm (m)	0.28 (0.03)	0.20/0.33	0.30 (0.02)	0.25 (0.02)	<0.001
V torso (dm ^3^)	37.00 (11.00)	28.00/61.00	44.71 (6.93)	27.00 (4.42)	<0.001
V upper arm (dm ^3^)	2.00 (0.80)	0.8/3.8	2.70 (0.67)	1.48 (0.38)	<0.001
V forearm (dm ^3^)	1.00 (0.30)	0.4/2.00	1.37 (0.20)	0.72 (0.16)	<0.001

*BMI, body mass index*.

Participants' body part masses and lengths were measured in accordance with statistical data (Plagenhoef et al., 1983), see Supplementary Figure S2 and Supplementary Table S1. The center of mass (COM) of the body segments were estimated according to the statistical data of the same author, for example, for males for the forearm, shoulder and the whole body, COM is equal 43, 43.6, and 63%, respectively and for females 43.4, 45.8, and 56.9 %, respectively.

### 2.3. Experimental Set Up

Direct and subjective methods were combined because they provided a larger range of parameters to understand a phenomenon. Direct methods involved the use of a timer, dynamometers (hand digital dynamometer (Camry scale store, North America) to measure hand muscle contraction, back-leg-chest (BLC) (Baseline, New York, USA) to measure leg-back muscle contraction and based on Bioelectrical impedance analysis (BIA) scale (Nokia Health, Body +, China) to determine body composition (percentage of body fat, water percentage, muscle and bone mass) of participants. In the subjective method, NASA-TLX was implemented.

**Figure 1:**
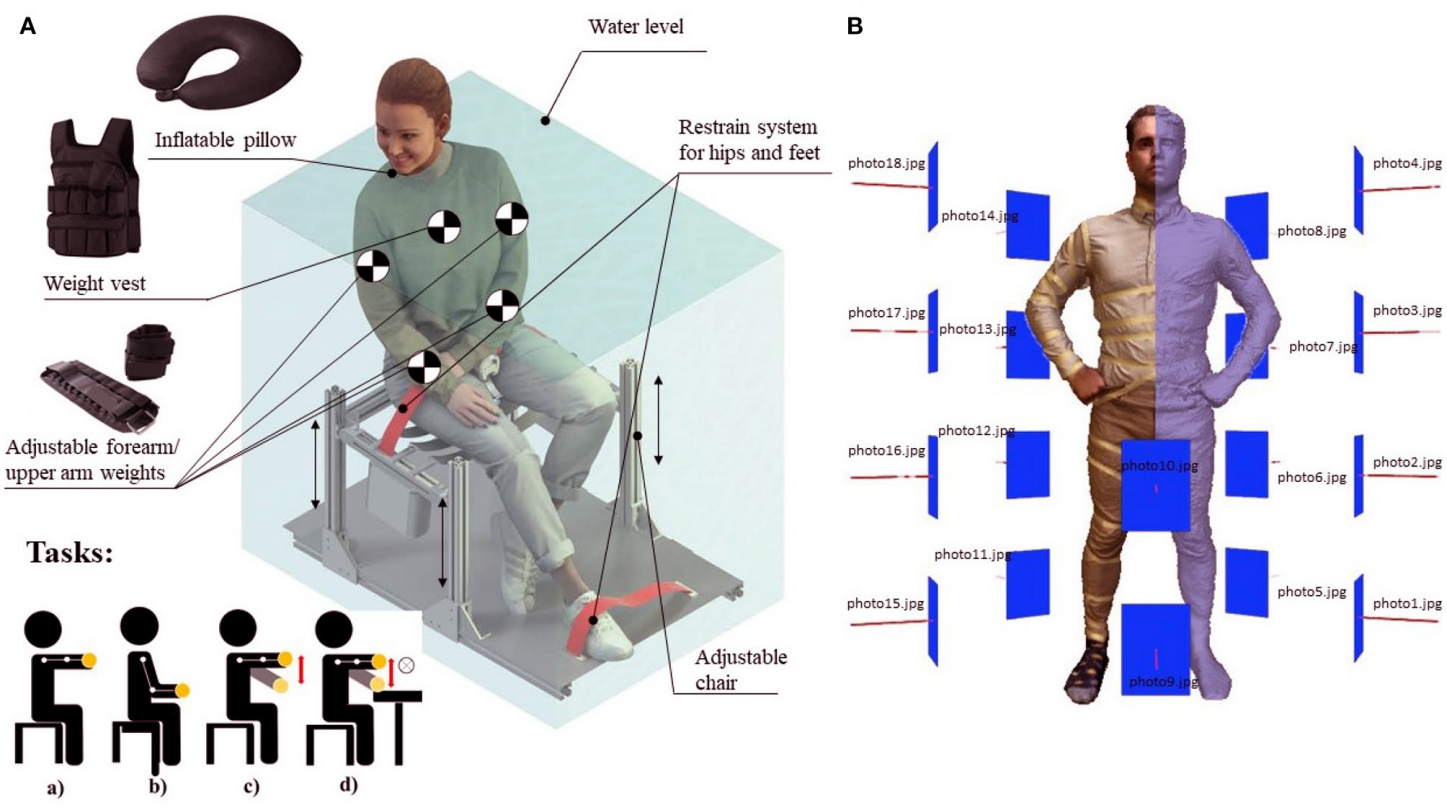
**(A)** Technical aspects of experimental setup. Tasks: a) Holding weight with an outstretched arm, Task (S1); b) Holding weights in an arm bent at the elbow, Task (S2); c) Slow 1 (lifting)-2 (lowering)-1-2 hand motion with weight (3 sec range of motion), Task (D); d) Repetitive motion with load 1(lifting)-2 (horizontal transfer)-3 (lowering)-4 (pause without load)-1-2-3-4 with constant repetition, Task (R). Image from: open source model https:/humano3D.com. **(B)** Participants 3D scan example with a visual explanation of the principle of photogrammetry (Agisoft Metashape).

First, a numerical model for the ballast calculation was developed, based on a simplified model of the human body. To assess the whole torso volume the photogrammetrie method was used due to the non-invasiveness for the participants in the experiment and the speed of measurements. However, in some cases, the reconstruction of the 3D volume of the hands required a lot of refinement and improvement of the mesh, and for this it was decided to measure this volume by the method of water displacement. To estimate the whole torso volume, the photogrammetrie method of the Agisoft Metashape 1.7.2 software (Agisoft LLC, St.Petersburg, Russia) was applied (Jebur et al., 2018). The bodies of all participants were photographed using a high-resolution camera (12 MP), focal length (4.25 mm), f/1.8, and 1,000 photographs were taken for each participant. All models were calculated with high or medium dense cloud quality and DSM 114 = 10 cm/Pixel resolution (see Figure 1B) First, a mesh of participants body created by photogrammetry was imported to Blender, called a modified mesh. Then a target mesh was added for each body segment, and a boolean modifier^1^ with difference option was applied to calculate the volume of a specific body segment (Freixas et al., 2006). The volumes of the participants were scaled and adjusted according to the height measured using a stadiometer (NutriActivia, Minnesota, USA). In some cases proportional editing^2^ (Guevarra, 2020) was required to modify irregular mesh. The Boolean method with a combination of proportional editing function allowed us to achieve a result that provides a difference in the estimate of the 3D model equal to 5 cm ^3^ for the whole body volume, compared to the water displacement method.

The volumes of the hand, forearm and shoulder were assessed as follow. First, hand up to wrist was immersed and a mark was made with the water level 1, then the hand was immersed up to the elbow and a new mark was made with the water level 2, and then the hand was immersed in the cylinder with water along the shoulder and a new mark wit ha level 3 was made. Then the difference between the water levels was calculated. The volume of the shoulder is equal to the difference in water displacement between the level 3 and 2 marked when the upper arm and elbow are immersed. The volume of the forearm was calculated based on the difference in water level 2 and 1 noted when the elbow and wrist were immersed. We neglected the volume of the wrist, because this volume is about less than 1% of the total body volume. This can be included in the overall error estimation.

Figure 1A shows experimental setup with a participant-adjusted chair. Electronics and sensors were not used in this study. All participants were strapped at hips and legs. The participant's legs were attached to the footrest in accordance with their anthropometry, taking into account the length of the legs from knee to foot in particular.

For experiments in the water tank, ballasts are added on different parts of the body of the participants. A weight vest (Thorn+Fit Schweiz, Bale, Switzerland) was used for the torso, and adjustable weights (Strength shop.ch, Switzerland) for the forearm and upper arm. The participants' upper limb and trunk ballast weights were calculated as follows:


(1) $$\begin{array}{l} m_{b.p}\cdot g_{m}=\left(m_{\text{b.p}}+m_{\text{b}}\right)\cdot g_{\text{e}}-\left(V_{\text{b.p}}+\frac{m_{\text{b}}}{\rho_{b}}\right)\cdot g_{e}\cdot \rho_{H2O} \end{array}$$

where *m*_b.p_ is the mass of the body part,

*V*_b.p_ - the volume of the body part,

*m*_*b*_ - the mass of the ballast,

*g*_*m*_ - the acceleration of gravity on the surface of the Moon,

*g*_*e*_ - the acceleration of gravity on the surface of the Earth,

ρ_*b*_ - the density of ballast weights,

ρ_*H*2*O*_ - the density of water.

Sand (ρ_*b*_ = 2816.9 kg/ m^3^) and lead (ρ_*b*_ = 11340 kg/ m^3^) and polystyrene (ρ_*b*_ = 30 kg/ m^3^) were used for ballast. For example for participant with trunk volume of 28.41 dm^3^, and mass of the trunk of 29.26 kg, ballast mass (lead) is 5.7 kg.

### 2.4. Tasks Description and Experimental Measurements

Four different one handed tasks were performed by the participants: holding weights with an outstretched arm (S1), holding weights in an arm bent at the elbow (S2), slow dynamic motion (D), and repetitive motion (R). Due to the fact that not 32 all participants were available for the same tasks, then 22 participants were invited for the task (S1), 27 participants conducted task (S2), 25 participants conducted task (D) and 26 participants conducted task (R) under 1G and ⅙ G. And only 6 participants completed similar tasks under ⅓ G condition. Six different task intensities were investigated 0.5, 1, 3, 5, 7, and 9 kg taking into account the corresponding level of gravity. The different intensities were different for each participant, especially the lowest and highest intensities. Lower intensities were tested less often because it was more time consuming and the participants were not always motivated to do monotonous work, and time was essential to conduct experiments with other intensities. Higher intensities were also tested less often since not all participants had the appropriate physical capabilities. The choice of maximum load was dependent on the physical capabilities of the participant and the gender (HSE, 2020). All task types, gravity and task intensity levels were randomized. All participants were asked to answer a questionnaire adapted for everyone to assess their readiness for physical activity (Warburton et al., 2019). It can be found in the Supplementary Figure S1. This questionnaire is an internationally renowned tool for participants screening. This questionnaire contained 7 questions related to general health and two options choices: “Yes” or “No.” Only those participants who answered "No" to all questions were cleared for physical activity and our experiments.

All participants in the experiment received detailed oral instructions to perform the tasks. More complex tasks, such as those involving repetition, were demonstrated in a video and each participant could consult video instructions throughout the exercise. All participants participated in warm-up exercises to be ready for physical activity. They performed a 3 kg dumbbell lift in their right hand for 5 min, 15 times with a break of 1 min. The tasks were first conducted in 1 G and then repeated in the water tank.

The right hand strength of each participant was measured with a hand digital dynamometer three times before and three times after the task. Then, the mean values were evaluated. The same approach was applied with a calibrated back-leg-chest (BLC) dynamometer. Due to the difficulty of the simulation of the tasks underwater, back measurements were not taken. These dynamometers provides kilograms (kg) and pounds (lb) estimates based on a certain amount of applied force. All measurements were taken in a seated position and took into account the position of the participant's limb (outstretched arm or arm bent at the elbow). For measurements with a BLC dynamometer, the length of the chain was adjusted to the participant's sitting height by asking the participant to sit on a chair and put their legs on the base of the BLC dynamometer, bent at 90 degrees. All tasks were performed until volitional failure. Between all tasks, participants were able to take micro-pauses (very short intermittent breaks) equal at least 1/5 of working time (Australia, 2011). In practice, rest breaks are largely the result of a worker's personal feedback of sufficient free time to allow workers to complete the activity with relative comfort (Brown, 1994). Therefore, if the participant needed more time to recover, then additional time was provided according to their individual needs. This time was not recorded by authors.

In an underwater environment, the speed of motions can play a crucial role and significant loads can occur from the water during fast movements. A rough estimate of this can be obtained by presenting the forearm of the participant, moving rectilinearly and evenly in the water, as an equivalent cylinder, and calculating the force of hydrodynamic resistance (*F*_*res*_). In this case, the diameter of the equivalent cylinder is:


(2) $$\begin{array}{l} d=2\cdot \sqrt{\frac{V_{b.p}}{\pi \cdot l_{b.p}}} \end{array}$$

where *l*_*b*.*p*_ is the length of the body part (or equivalent cylinder), Then the resistance force:


(3) $$\begin{array}{l} F_{res}=C_{x}\cdot S\cdot \frac{\rho_{H2O}\cdot V^{2}}{2} \end{array}$$

where *S* = *l*_*b*.*p*_·*d*- flow obstruction area,

*C*_*x*_ = 0.5 - drag coefficient (Savitsky, 1972),

*V*- the speed of the object in a fluid.

The limiting speed of the forearm was calculated from the condition that water resistance force did not exceed 10% of the weight of the forearm: *F*_*fa*_ = *m*_b.p_·*g*_e_. In this case speed of the forearm should be less than 47 cm · s^−^1. The range and speed of the participant's dynamic motions were designed in accordance with the found value and were approximately 10 cm · s^−^1 for a 3-s motion cycle. This means that we had a margin equal to 3.

### 2.5. NASA-TLX

The NASA-TLX scale was originally developed for the aviation industry. Then this scale was applied to power plants, remote control systems, and space applications. Different human factors were assessed with this approach: team collaboration (6%), fatigue (2%), tensity (3%), experience (4%), and disability (1%) (Hart, 2006).

In our study, the NASA-TLX (Hart and Staveland, 1988) scale was applied to assess the mental workload of the participants. This assessment was carried out immediately after the execution of each task under 1G and simulated ⅙ G, as well as ⅓ G. The benefit of this approach is that it reveals a specific demand of each participant.

NASA-TLX is based on independent subjective demands related to: Mental (MD), Physical (PD), and Temporal Demands (TD), Performance (P), Effort (EF) and Frustration (FR). Before completing the task, the participants were asked to read the detailed description of these subjective demands, see Supplementary Figure S3. NASA - TLX consisted of two parts. The first part is based on individual weighing of subjective demands through 15 pairwise weighing. Participants were asked to select the most appropriate subjective demand for the workload from each pair. The 15 pairs for pairwise comparison included: MD or PD, MD or EF, PD or FR, PD or P, PD or TD, FR or MD, FR or EF, P or MD, P or TD, EF or PD, EF or P, TD or FR, TD or MD, TD or EF, P or FR. The total number of selected specific subjective demands is called task load index or *Weight*_*demand*_. The calculation of which will be discussed below. The second part is based on 100 point range *Rating*_*demand*_. For this evaluation the following questions for all subjective demands were asked (Hart, 2006):

- MD - How mentally demanding was the task?
- PD - How physically demanding was the task?
- TD - How hurried or rushed was the pace of the task?
- P - How successful were you in accomplishing what you were asked to do?
- EF - How hard did you have to work to accomplish your level of performance?
- FR - How insecure discouraged, irritated, stressed, and annoyed were you?

Then each subjective demand of participants was weighted, resulting in a composite mental workload, see Equation 4. The calculation of overall mental workload is as follows:


(4) $$\begin{array}{l} WWL=\frac{\sum \left(Weight_{demand}\cdot Rating_{demand}\right)}{15} \end{array}$$

where *Weight*_*demand*_ - task load index based on pairwise comparison of subjective demand (total 15 pairs) *Rating*_*demand*_ - a total score of 0 corresponds to a very low subjective demand, a total score of 100 corresponds to a very low subjective demand.

## 3. Results

### 3.1. Fatigue Curves for 1G, ⅙ G

The following dataset were combined in an Excel: sample size, sex (male, female), mean age, ET (min), task intensity in kg and in newtons with respect to 1G, ⅙ G and ⅓ G, muscle voluntary contraction (hand and leg-spine) measured before and after each task, mental workload data. The main descriptive statistics of the participants with *p*-value calculation are presented in Table 1. The males were significantly taller, heavier than the females (*p* < 0.001).

First, we built curves for different gravity conditions (1G and ⅙ G). Each participant's ET (min) is normalized for the participants' ratio of muscle mass (kg) and body mass (kg).

**Figure 2:**
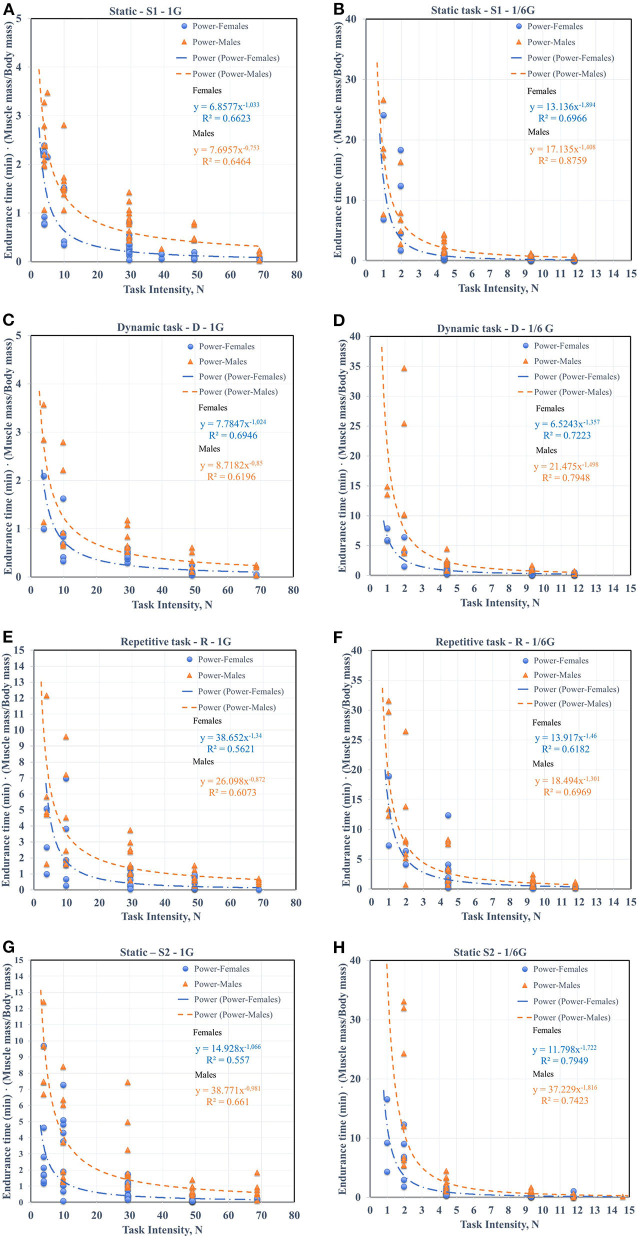
The ET power models (*N* = 520 studies, 6 task intensities, **(A)** static task (S1)-1G, **(B)** static task (S1) ⅙ G, **(C)** dynamic task (SD)-1G, **(D)** dynamic task (D)-⅙ G, **(E)** repetitive task (R)-1G, **(F)** repetitive task (R)-⅙ G, **(G)** static task (S2)-1G, and **(H)** static task (S2)-⅙ G.

Task Intensity is expressed in Newtons for 6 different loads (0.5, 1, 3, 5, 7, and 9 kg) taking into account the corresponding level of gravity. We defined two trends in the data set corresponding to females and males. The power model has superior fit over exponential fit all data for each task and specific environment due to slightly greater R^2^, as seen in Figures 2A–H. Thus, we used power models for 1G and ⅙ G comparisons. Then the constants *b*_*o*_, *b*_1_, and R^2^ values of power trendline equation for all models are provided in Table 2.

**Table 2:** Task Intensity-Endurance time power model.

**Power model**	***b*_*o*_**	***b*_1_**	***R* ^2^**	**mn ET (min)**	**mn ET (min)**	**mn ET (min)**
				**load (1 kg)**	**load ( 3 kg)**	**load (5 kg)**
			22 participants			
S1-1G	7.70/6.86	–0.75/–1.03	0.65 / 0.66	1.67/0.95	0.85/0.34	0.39/0.14
S1-⅙ G	17.13/13.14	–1.40/–1.89	0.87/0.70	7.73/6.21	2.65/0.65	0.64/0.30
			25 participants			
D-1G	8.72/7.79	–0.85/–1.02	0.62/0.69	1.30/0.80	0.79/0.35	0.28/0.08
D-⅙ G	21.47/6.52	–1.50/–1.36	0.79/0.72	14.93/9.34	2.16/0.82	0.77/0.34
			26 participants		
R-1G	26.10/38.65	–0.87/–1.34	0.61/0.56	6.39/2.54	1.93/0.72	0.75/0.21
R-⅙ G	18.49/13.92	–1.30/–1.46	0.70/0.62	9.80/5.31	4.43/2.84	1.06/0.49
			27 participants			
S2-1G	38.77/14.93	–0.98/–1.07	0.66/0.56	2.43/0.65	0.80/0.69	0.80/0.16
S2-⅙ G	37.22/11.80	–1.81/–1.72	0.74/0.79	15.72/6.63	2.38/0.94	0.82/0.29
			6 participants	(M/F)		
S1-1G	1.56	–1.19	0.77	1.43	0.56	0.22
S1-⅓ G	2.71	–1.32	0.72	2.62	0.77	0.32
S1-⅙ G	7.89	–1.55	0.84	10.34	1.29	0.60
D-1G	1.57	–1.03	0.56	1.70	0.62	0.32
D-⅓ G	3.38	–1.34	0.80	3.70	0.92	0.37
D-⅙ G	5.49	–1.27	0.77	6.57	1.60	0.68
R-1G	5.25	–1.85	0.57	3.15	0.89	0.33
R-⅓ G	6.64	–1.37	0.60	6.24	1.39	1.08
R-⅙ G	10.20	–1.38	0.71	9.69	2.06	1.00
S2-1G	2.51	–0.94	0.52	4.26	1.20	0.51
S2-⅓ G	6.12	–1.46	0.82	6.36	1.28	0.55
S2-⅙ G	7.66	–1.47	0.85	9.55	1.37	0.69

*Power (ET = $b_{\text{0}}\cdot {\left(TaskIntensity\right)}^{b_{\text{1}}}$) model with coefficients b_0_ and b_1_. The results are presented first for males and then for females in the first four groups and mixed males and females for 6 participants. Task types: holding weights with an outstretched arm (S1), holding weights in an arm bent at the elbow (S2), slow dynamic motion (D), and repetitive motion (R). Task intensity is presented for 1, 3, and 5 kg (⅙ G)*.

According to the results, Coefficients *b*_*o*_ vary greatly, while coefficients *b*_1_ have quite similar values for 1G and ⅙ G for males and females. The average values of the coefficient *b*_1_ = 0.86 for males and *b*_1_ = 1.11 for females for 1G, while the average values of *b*_1_ = 1.50 for males and *b*_1_ = 1.61 for females for ⅙ G.

All models for all tasks have the average value *R*^2^ = 0.63 for males and average value *R*^2^ = 0.62 for females for 1G and average *R*^2^ = 0.77 for males, average value *R*^2^ = 0.70 for females conducting the tasks under ⅙ G. The lower values of *R*^2^ for 1G are most likely due to the fact that a smaller number of participants could work with loads of 5 and 7 kg. For example 80% of females couldn't work with 5–7 kg load under 1G.

Consistent with all curves, ET (min) increased for simulated lunar gravity in comparison with 1G. We found the average ET (min) for 1, 3, and 5 kg for all types of tasks to identify the growth rate. For a static task (S1) with a load of 1 kg, the ET of male increased 4.62 times and 6.53 times for female for ⅙ G compared to 1G. For the same task with a load of 3 kg, the ET of male increased 3.11 times and 1.91 for female for ⅙ G compared to 1G. And for load of 5 kg, the ET of male increased 1.64 times and 2.14 for female for ⅙ G compared to 1G. As can be seen from the given example, with increasing load, the ratio between ET (min) under ⅙ G and 1G decreases.

#### 3.1.1. Hand and BLC Muscle Contraction

In Table 3 you can see the average values for all task intensities from 0.4 to 9 kg for hand (H) and BLC strength measured for each participant before and after each task in an appropriate environment, 1G, ⅓ G and ⅙ G. The percentage change in values after each task is calculated for hand and BLC muscle contraction to compare the results.

**Table 3:** Before and after variation hand (H) and back-leg-chest (BLC) strength values, including % of variation.

**G-level**	**H (Before)**	**H (After)**	**Δ (%)**	**BLC (Before)**	**BLC (After)**	**Δ(%)**
**Task**	**Mean (SD)**	**Mean (SD)**	**H**	**Mean (SD)**	**Mean (SD)**	**BLC**
			22 participants		
1G-S1	48.84 /24.95	48.95/24.66		7.81 /2.83	7.89 /2.43	
	(2.92)/(1.82)	(2.05)/(2.09)	0.21/–1.21	(0.83)/(0.16)	(0.82)/(0.22)	0.99/–16.12
⅙ G-S1	47.30/26.28	45.86/24.82				
	(1.12)/(3.53)	(1.46)/(2.78)	–4.95/–4.91	NA	NA	NA
			25 participants		
1G-D	46.02/22.15	45.49/20.84		8.03/2.55	8.01/2.18	
	(7.57)/(1.09)	(5.73)/(1.50)	–1.17/–6.29	(1.34)/(0.25)	(1.36)/(0.41)	–0,19/–16.41
⅙ G-D	46.37/24.46	44.96/24.91				
	(2.61)/(5.10)	(1.13)/(5.10)	1.83/–3.14	NA	NA	NA
			26 participants		
1G-R	48.47/23.79	47.41/22.65		8.57/2.88	8.13/2.65	
	(4.18)/(3.41)	(3.65)/(2,33)	–2.23/–5.04	(0.86)/(0.51)	(0.88)/(0.59)	–5.47/–8.87
⅙ G-R	44.00/22.96	43.75/22.15				
	(5.44)/(3.02)	(5.22)/(2.85)	–0.58/–3.64	NA	NA	NA
			27 participants		
1G-S2	44.56/20.52	42.49/19.48		13.46/5.15	12.73/4.75	
	(3.52)/(1.21)	(2.39)/(0.71)	–4.87/–5.37	(1.22)/(1.49)	(1.61)/(1.36)	–5.73/–8.49
⅙ G-S2	43.27/22.03	41.70/20.67				
	(2.84)/(2.05)	(1.48)/(2.24)	–3.77/–6.61	NA	NA	NA
			6 participants		
1G-S1	53.05/26.05	49.12/25.94	–8.00/–0.41	NA	NA	NA
⅓ G-S1	51.02/23.09	49.02/21.88	–4.09/–5.55	NA	NA	NA
1G-D	47.85/26.57	47.81/26.55	–0.08/–0.08	NA	NA	NA
⅓ G-D	51.04/25.07	49.55/24.51	–3.02/–2.29	NA	NA	NA
1G-R	51.22/27.42	46.93/24.50	–9.13/–11.91	NA	NA	NA
⅓ G-R	48.76/25.57	46.65/24.89	–4.53/–2.76	NA	NA	NA
1G-S2	46.76/24.76	42.63/22.29	–9.68/–11.10	NA	NA	NA
⅓ G-S2	48.75/23.42	47.15/22.89	–3.39/–2.33	NA	NA	NA

*The results are presented first for males and then for females for all cases. Task types: holding weights with an outstretched arm (S1), holding weights in an arm bent at the elbow (S2), slow dynamic motion (D), and repetitive motion (R). Analyzed task intensities: 0.5, 1, 3, 5, 7, and 9 kg, respectively. All values are presented as mean and standard deviation (SD). NA-the values that could not be measured for logistic reasons of the experiment*.

Estimated averages of muscle contraction force indicate a greater reduction in physical strength under ⅙ G than 1G. This is consistent with the fact that the participants were able to work longer and their ET (see Table 3) is higher in a simulated ⅙ G environment due to lower loads and the weight of the participants themselves. Overall, all participants showed a greater decrease in hand strength after doing all tasks under 1G and ⅙ G. The same pattern is seen for the BLC measurements. There is very rarely an increase in muscle contraction after the tasks. And this may be due to non-compliance by participants with the instructions for using anemometers or the individual characteristics of the participants.

### 3.2. NASA-Task Load Index for 1G, ⅙ G

To investigate the mental workload of participants in 1G and ⅙ G, overall mental workload (WWL) in %, as well as average values of MD, PD, TD, P, EF, FR are calculated with NASA-TLX, see Table 4. In this table we gave one example of a study of the effect of 3 kg load on participants' mental workload. The results of the subjective questionnaire taking into account the responses collected in the ⅓ G simulation with 6 participants are included in the same table.

**Table 4:** Summary of the calculated NASA-TLX parameters for the tasks with 3 kg load.

**Task**	**MD**	**PD**	**TD**	**P**	**EF**	**FR**	**WWL(%)**
**G-level**	**mean**	**mean**	**mean**	**mean**	**mean**	**mean**
			22 participants		
S1-1G (M)	40.72	275.54	71.09	118.63	232.91	56.45	53.02
S1-1G (F)	49.77	264.69	102.92	101.08	252.61	49.08	54.67
S1-⅙ G (M)	42.70	198.10	94.50	163.90	181.30	10.4	46.6
S1-⅙ G (F)	75.00	226.67	145.78	120.67	179.44	8.11	50.37
			25 participants		
D-1G (M)	47.00	279.60	30.10	148.50	286.00	87.10	58.55
D-1G (F)	35.20	303.80	64.90	105.70	204.60	14.30	48.57
D-⅙ G (M)	73.00	187.90	82.00	108.90	134.60	0.40	39.12
D-⅙ G (F)	12.25	231.25	107.75	143.75	214.50	21.50	48.73
			26 participants		
R-1G (M)	115.00	190.22	132.11	130.89	197.89	9.89	51.73
R-1G (F)	48.09	285.09	75.45	92.73	194.45	44.64	49.36
R-⅙ G (M)	92.50	167.00	106.50	126.00	158.50	6.00	43.77
R-⅙ G (F)	54.30	215.80	142.10	135.80	184.20	39.40	51.44
			27 participants		
S2-1G (M)	58.08	247.46	88.69	119.46	165.54	48.38	48.51
S2-1G (F)	58.92	318.58	125.58	118.42	272.75	12.92	60.48
S2-⅙ G (M)	57.89	184.89	66.67	91.33	181.44	13.56	39.72
S2-⅙ G (F)	59.64	269.64	127.00	151.00	211.36	11.64	55.35
			6 participants		
S1-⅙ G (M,F)	40.92	129.92	121.25	72.50	115.42	26.00	33.73
S1-⅓ G (M,F)	31.36	150.90	89.09	147.27	142.27	40.90	40.12
S1-1G (M,F)	21.91	278.73	54.64	111.73	149.45	16.91	42.22
D-⅓ G (M,F)	61.25	114.17	53.75	90.42	143.33	38.42	33.42
R-⅓ G (M,F)	44.09	118.18	75.45	105.45	146.82	51.82	36.12
S2-⅓ G (M,F)	49.50	97.00	68.50	74.50	130.00	40.80	30.69

*The results are presented first for males and then for females in the first four groups and mixed males and females for 6 participants. Task types: holding weights with an outstretched arm (S1), holding weights in an arm bent at the elbow (S2), slow dynamic motion (D), and repetitive motion (R). Analyzed Task Intensities: 0.5, 1, 3, 5, and 7 kg. All values in table are means. Subjective demands were multiplied by task load index, thus some parameters are higher than 100 range due to weighting parameters*.

In our study, we found that all average values of WWL,% are lower for all type of tasks for males and females for simulated ⅙ G compared to to the data obtained under 1G. We found 12% decrease in average value of WWL,% for static task (S1), 33% for dynamic task (D), 15% for repetitive task (R) and 23% for static task (S2) for males under ⅙ G vs. 1G. The average WWL,% values decreased for static task (S1) by 8%, for dynamic task (D) the values remained the same, for the repetitive task (R) the values increased by 4 % and for static task (S2) increased by 12% for females in ⅙ G compared to 1G. It is important also to analyse the impact the different demands of the overall workload. In accordance with all presented data, physical demand (PD) and effort (EF) have the highest values for males and females. It was also observed that WWL,% is systematically higher for females performing tasks under ⅙ G than for males, Table 2.

### 3.3. Comparison 1G, ⅙ G, ⅓ G

**Figure 3:**
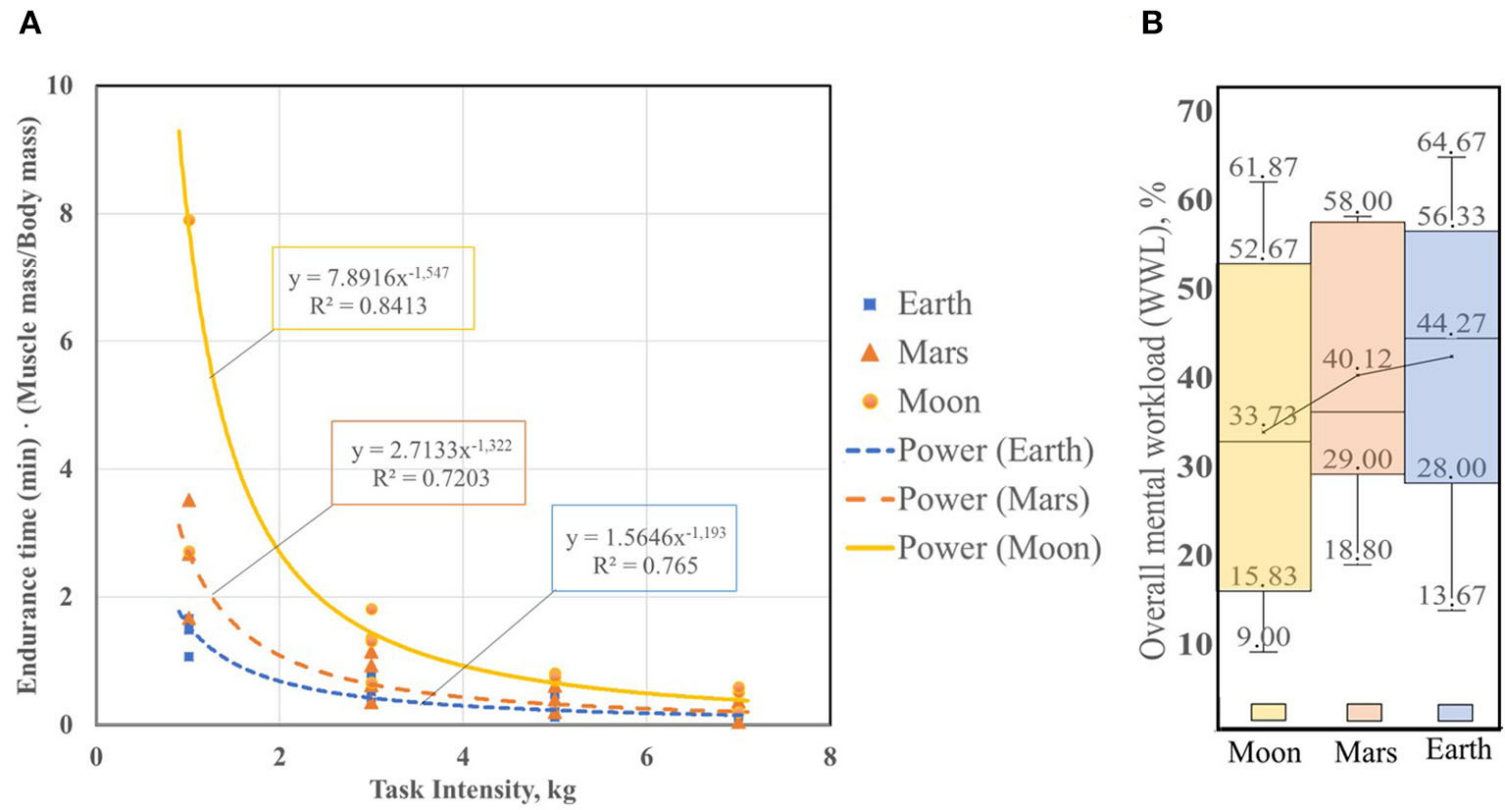
**(A)** Endurance time-gravity level dependence for static tasks for Earth, simulated Moon and Mars gravity levels. Task Intensity in kg. **(B)** Overall mental workload (WWL,%) [example for static task (S1)], for males and females for loads (1, 3, 5, and 7 kg)-gravity level dependence.

Due to the experimental conditions and the need to use large ballast weights, which may be invasive for the participants, a small number of participants were invited (3 males and 3 females). We had only a small amount of data to carry out a comparative analysis of the effect of gravity on participant's fatigue in ⅓ G doing static, dynamic and repetitive tasks. Figure 3A shows an example of the fatigue curves for 1G, ⅙ G and ⅓ G, where the fatigue curve for simulated lunar gravity is located above the curve for Martian gravity and Earth gravity. We built only one power trend curve due to this limited data, and we took into account the intensity of the task from 1 to 7 kg. The values of *b*_*o*_, *b*_1_, *R*^2^ mean muscle mass ET ratio of muscle mass (kg) and body mass (kg) of the participant for 1 kg load, 3 kg and combined 5 and 7 kg loads for all types of tasks are presented in the Table 2.

We found the ratio between the mean ET (min) values for 1, 3, and 5–7 kg loads, normalized to the participants' ratio of muscle mass (kg) and body mass (kg) for ⅙ G and 1G, and then for ⅓ G and ⅙ G. For static tasks (S1), the ratio between ET (min) for ⅙ G and 1G is 4.10, and the ratio between ET(min) for ⅓ G and 1G is 1.60. For dynamic tasks (D) the ratio between ET(min) for ⅙ G and 1G is 2.87, and the ratio between ET (min) for ⅓ G and 1G is 1.25. For repetitive tasks (R), the ratio between ET(min) for ⅙ G and 1G is 2.82, and the ratio between ET (min) for ⅓ G and 1G is 2.30.

For a more complete understanding of the phenomenon, we also conducted a NASA-TLX survey after the participant completed the task. Figure 3B shows the Box-and-whisker plots with outliers for Earth, simulated lunar and martian gravity. For static tasks we see the normal distribution for ⅙ G, ⅓ G and 1G. According to the respective median values for each environment and each box plot we can say that there is little difference between the three groups of data, but there is nonetheless a tendency for the workload to increase with increasing gravity. We found 10.54% increase of average values of WWL,% for static task for males and females under 1G vs. ⅙ G. And we found 4.15 % increase of average values of WWL,% for static task for males and females under 1G vs. ⅓ G.

The results of dependence between ET (min) and WWL,% and muscle contraction from the gravity level (1G, ⅓ G and ⅙ G), as well as the character of this dependence are presented in Table 5. It shows the ET (min) and WWL% predictors for Static (S1) and dynamic (D) tasks under 1G, ⅓ G and ⅙ G for males and females.

**Table 5:** The ET (min) and WWL% predictors of different types of tasks under 1G, ⅓ G, ⅙ G.

**Task**	**ET (min)**	**WWL%**	**(H) before (kg)**	**(H) after (kg)**
**G-level**				
		n = 131		
S1 (M/F)	–0.25 (0.08)^**^	0.03 (0.02)^*^	–0.22 (0.12)^*^	0.24 (0.13)^**^
		*n* = 102		
D (M/F)	–0.33 (0.11)^**^	0.04 (0.02)^**^	–0.09 (0.11)	0.08 (0.12)
		*n* = 115		
R (M/F)	–0.08 (0.06)	0.00 (0.02)	–0.14 (0.14)	0.01 (0.15)
		*n* = 237		
S2 (M/F)	–0.09 (0.05)^**^	0.00 (0.01)^**^	0.03 (0.09)	–0.01 (0.09)

*All results are mixed for Males (M) and Females (F). Task types: holding weights with an outstretched arm (S1), holding weights in an arm bent at the elbow (S2), slow dynamic motion (D), and repetitive motion (R)*.

** *p < 0.05*,

* *p < 0.10. Robust standard errors in parentheses*.

These results indicate that HG increases a participant's productivity by reducing overall physical fatigue expressed in ET (min) compared to Earth's gravity. This was confirmed by a defined significant positive (p=0.002) relationship between Endurance time and gravity level (⅙ G, Moon, ⅓ G, Mars, 1G) with negative coefficient for male and female participants for a static task. This means that increasing gravity will reduce ET (min).

There is a significantly positive relation (*p* < 0.05) between ET (min) and gravity levels (1G, ⅓, ⅙) with a negative coefficient of correlation equal (–0.25, –0.33, and –0.09) for all tasks except repetitive one. At the same time there is a moderate relation (*p* < 0.1) for static (S1) task and significantly positive relation (*p* < 0.05) for static (S2) and dynamic (D) tasks between WWL,% and gravity levels (1G, ⅓, ⅙) with a positive coefficients of correlation. We found a significant relation (*p* < 0.05) between hand muscle contraction force and gravity level only for static task (S1).

## 4. Discussion

This is the first study to provide insight into the effect of HG (⅙ G and ⅓ G) on data such as ET (min) of the upper extremities of participants and their mental workload. In addition, this study contributes to a clearer understanding of the relationship between physical fatigue of the upper limbs and mental workload when participants perform tasks under HG.

The results indicate that HG increases a participant's productivity by reducing overall physical fatigue expressed in ET (min) compared to Earth's gravity. This was confirmed by a defined significant positive (*p* = 0.002) relationship between Endurance time and gravity level (⅙ G, Moon, ⅓ G, Mars, 1G) with negative coefficient for male and female participants for a static task. This means that increasing gravity will reduce ET (min).

Our results also show a general decrease in overall mental workload, WWL,% under the same conditions. This is due to a decrease in PD and EF demands multiplied by task load index under ⅙ G in comparison with 1G and lower frustration values for males and females. It was also observed that WWL,% is systematically higher for females performing tasks under ⅙ G than for males, because most subjective demands are also higher for females, with the exception of P, EF and FR for the tasks (S1) and MD for the tasks (D) and (R). We assume that this is due to the female's higher sensitivity to the loads and weaker physical strength.

This study shows a moderate relation (*p* < 0.1) between overall mental workload and gravity level with a positive coefficient for male and female participants for the static task (S1), see Table 5. Lower p-values for WWL,% may be related to each participant's individual understanding and interpretation of the survey. Thus, increasing gravity will increase the mental workload. Other variables, such as hand muscle contraction after task, also had significant relation (*p* < 0.05) and positive correlation (0.024) with gravity level. The same trend was observed for dynamic and repetitive tasks.

These results could be applied to practical application for astronauts training for missions to the Mons and Mars, because by defining the level of physical fatigue in specific environments we can develop guidelines with respect to defined capacities of males and females. And these correlations may suggest that NASA-TLX assessment can be an appropriate tool for preliminary studies of mental workload, independently or in combination with other tools.

With all our participants we found that power function better matched the data of ET (min) - Task Intensity, but without specification of upper limb joints. This is similar to such studies as Rohmert (1960), Monod and Scherrer (1965), Huijgens (1981), Sato et al. (1984), Rohmert et al. (1986), and Sjøgaard (1986). The exponential model was used by the following authors Manenica (1986), Matthijsse et al. (1987), and Rose et al. (2000). The power model was chosen because of better fit of curve to the data and higher R^2^. We also subsequently found that the data for males and females should be divided into separate data sets because it results in a better curves fit and higher R^2^. This can be related to the different physical capacities of the male and female participants as well as their anthropometry.

The main finding of the study with 24 participants conducting Static task (S1) was related to three variables: ET (min), mental workload and contraction force. ET increased by an average of 3.54 times for females and 3.14 for males under ⅙ G, in comparison with 1G. Another interesting finding is related to the ratio value between the average ET (min) values for loads of 1, 3, and 5–7 kg, normalized to the ratio of muscle mass (kg) and body mass (kg) of participants, measured under ⅙ G and 1G. This is systematically higher for females than for males, see Table 2. For example, for male for the task (S1) with an intensity of 1 kg this ratio is 4.62 and for females is 6.54. This may confirm that females in general are more fatigue resistant and have a faster recovery during prolonged intense activity. Conversely our results suggest that males have a higher maximal power output.

We assume that it is also due to the higher sensitivity of females to loads, especially under 1G, due to skeletal muscle function. For example, while most males are able to perform the task with a load of 5 kg, whereas few females can complete it, and accordingly there is a large gap in ET results. If under 1G and underwater measurements of ET (min) for males are on average two times higher than for females, there will be a greater difference for females. Similar trends and relations were found for the dynamic (D), repetitive (R) and static (S2) tasks.

With the same 24 participants we found that under ⅙ G, mental workload reduced by an average 1.15 times for males and 1.08 for females in comparison with 1G for the static tasks (S1). We found that PD and EF demands have the highest impact on the overall mental workload which is consistent with Brown (1994). According to Xu et al. (2018), the "control of movement is a kind of mental activity that can cause mental fatigue," because the participant must make more effort to complete the task after increasing physical fatigue. Even if according to Rubio et al. (2004) NASA-TLX highly correlates with performance and according to our results it increases with reduction of the gravity level, the physical demand and effort significantly reduced with reduction of the gravity level.

Regarding assessment of muscle contractions of the hand before and after the tasks, a higher change in % for ⅙ G than for 1G was observed. This is because, in general, all participants worked longer at ⅙ G and became weaker in terms of upper limb strength. It should be noted, however that back-leg-chest measurements were not collected due to logistic reason.

With six participants we found that under ⅓ G, ET increased by a factor of 1.60 and ET increased by a factor of 4.10 and mental workload decreased by a factor of 1.26 for males and females in comparison with 1G. The same trend was found for the dynamic (D), repetitive (R) and static (S2) tasks. Although there is a pattern of increasing ET (min) with decreasing gravity, these results do not fully converge with those obtained in the experimental group with more participants, because of size effect. To increase reliability of the data, an experiment with a larger number of participants with a simulation of reduced gravity and the same tasks is recommended. Nevertheless, it is critical to have the third environment ⅓ G with an additional gravity level for statistical predictions for different effect studies.

The state of upper extremities under HG has in general been studied very little in comparison with lower extremities.The most of the studies focused on running, hopping and jumping under HG (Lacquaniti et al., 2017; Richter et al., 2017, 2021; Weber et al., 2019). G-level effect was most significant on peak planar force, gait cycle duration, pace and mechanical work in the HG conditions reduced compared to 1G. Additionally, reduced gravity below 0.4 G is insufficient to support musculoskeletal and cardiopulmonary systems for a long period of time (Richter et al., 2017). Another study Lauer et al. (2018) showed that water reduces the mechanical load on the shoulder by up to 75%. This is also seen in our findings: since all the movements were performed in water, even though each participant was given additional ballasts to make the body heavier, the total body weight still remained 6 or 3 times (⅙ G, ⅓ G, respectively) lighter than under 1G.

While previous research has focused on lower extremities under HG, our results demonstrate that the study of upper extremities is also important and will play a crucial role for short term and long-term missions and regular work on the Moon. These results should be taken into account when considering how to design manual tasks, including astronaut training tasks for specific environments under HG. The motions with extension and ulnar deviation should be investigated during future experiments.

In parallel and in combination with posture studies, it can be also scaled to working space ergonomics under HG in general. With this data we will be able to design and optimize workplace and manual operations. Such design will require less effort due to physical fatigue optimization. Also, this has potential for reducing repetitive strain injury and muskuloskeletal disorders which are commune in many workplace situations and contribute to absenteeism and additional costs for workforce.

The developed model could be used to assess physically limiting situations in industry in 1G and HG to propose alternative solutions. Furthermore, we recommend applying it to digital human modeling, which requires experimental data for modeling and further predictions. Consequently, this will lead to the development of new guidelines and standards for workplace design under HG.

## 5. Conclusions

This paper proposes an empirical and a subjective model for physical fatigue of upper extremity fatigue and mental workload assessment with three levels of gravity, six task intensities, and four types of tasks. These new models show excellent agreement between experimental data and subjective data.

With ET (min) assessment, we found that participant performance reduced with increased gravity level for all types of tasks. With mental workload assessment, we found that the workload in ⅙ G is lower than in 1G for the same tasks. In our additional test with comparison of impact of 1G, ⅓ G, ⅙ G on six participants' physical strength we found constituency and a certain linearity, expressed by increasing the physical fatigue and workload with gravity level increasing. According to the small sample we can see that for all tested tasks the level of physical fatigue and mental stress for the simulated gravity of Mars is between levels estimated during the experiments under 1G and ⅙ G. It can be certainly be generalized.

Our results could be integrated into digital human simulations, helping to carry out longer-period simulations, for example, over years. If we can measure productivity in such an environment, we can improve the workplace design and develop a new hypothesis.

We recommend using an empirical fatigue model with a subjective assessment tool. It provides a better understanding of the phenomena and suffices to predict the fatigue curves for a particular task. Application of subjective mental workload assessment can be critical for workplace equipped with human-machine systems designed to ensure higher levels of comfort, performance, and safety.

## 6. Limitations

Although some valuable findings were obtained, there are still several limitations to this study. The main limitation arises from variations in the age, anthropometry and conducting asymmetric tasks of the participants. In order to determine these effect, younger and older, weaker, stronger populations, should be analyzed in future studies. As well as symmetric tasks should be investigated. In addition, we recommend validating ⅓ G fatigue curve with a larger number of participants. Finally, we recommend validating all defined empirical and subjective models with parabolic flights adapted to specific environments. The study of the fatigue effects on the posture of the participants may be a new attempt to explore the participant's fatigue states.

## Data Availability Statement

The datasets presented in this study can be found in online repositories. The names of the repository/repositories and accession number(s) can be found at: https://figshare.com/s/03b02a630edfde797a12.

## Ethics Statement

The studies involving human participants were reviewed and approved by Human Research Ethics Committee-EPFL [HREC 507 024-2021/09.03.2021 amendment to initial protocol HREC 001-2020/20.12.2019)]. The patients/participants provided their written informed consent to participate in this study.

## Author Contributions

TV organized the experiments, invited participants, collected relevant data during experiments, performed the statistical analysis, processed data, and drafted manuscript. CN an ESA astronaut, provided space-related expertise for the preparation of the experiments, helped coordinate the experiments, and draft the manuscript. VG participated in the design and coordination of the study, helped to draft the manuscript, and provided the founding for this study. All authors contributed to the article and approved the submitted version.

## Funding

The manuscript was funded by Space Innovation and EPFL Library's Open Access fund.

## Conflict of Interest

The authors declare that the research was conducted in the absence of any commercial or financial relationships that could be construed as a potential conflict of interest.

## Publisher's Note

All claims expressed in this article are solely those of the authors and do not necessarily represent those of their affiliated organizations, or those of the publisher, the editors and the reviewers. Any product that may be evaluated in this article, or claim that may be made by its manufacturer, is not guaranteed or endorsed by the publisher.

## Footnotes

^1^**Boolean modifier** allows to perform logical operations with complex 3D meshes. These operations include intersect, difference and union options. Difference option allows to subtract the target mesh from the modified mesh so that anything outside the target mesh is retained.

^2^**Proportional editing** is a way of modifying selected model elements (such as mesh elements) with a scaling effect. This method is convenient for smooth deformation of the mesh surface.
