# Supplementary material for: An Empirical and Subjective Model of Upper Extremity Fatigue Under Hypogravity
Source: Front Physiol. 2022 Feb 16;13:832214. doi: 10.3389/fphys.2022.832214 (PMC8888417; doi:10.3389/fphys.2022.832214)
Supplement: Supplementary file 1 [file Data_Sheet_1.pdf]

Supplementary Material

1 SUPPLEMENTARY TABLES AND FIGURES

Supplementary materials include information related to the size effect of the experiments, anatomical data, general health questions and the subjective NASA Task Load Index (NASA-TLX) assessment of mental workload.

1.1 Power analysis and Size effect

1.1.1 Required number of participants

Our experiment was planned for 4 independent groups, each undergoing 2 different conditions. G*Power 3.1.9.7 was used to calculate the necessary total sample size (power analysis) to observe an average size effect with a Type I error rate of 0.05 (α=0.05) and a power of 0.95 (1-β=0.95) for: i) the effect of the interaction between group and condition; ii) for the effect of group; iii) and for the effect of condition. We expect the results from different conditions to be somewhat correlated (r=0.4) and assume that sphericity assumptions are met.

Results for the power analysis for the interaction term shows that total of 76 subjects (to be split evenly between 4 groups) are required to observe a significant average size interaction with a Type I error rate of 0.05 (α=0.05) and a power of 0.95 (1-β=0.95).

Results for the power analysis for the effect of Group shows that a total of 132 subjects (to be split evenly between 4 groups) are required to observe a significant average size interaction with a Type I error rate of 0.05 (α=0.05) and a power of 0.95 (1-β=0.95). If we aim for a power of 0.8 (1-β=0.8), 82 subjects would be necessary.

Results for the power analysis for the effect of Condition shows that a total of 36 subjects are required to observe a significant average size interaction with a Type I error rate of 0.05 (α=0.05) and a power of 0.95 (1-β=0.95).

1.1.2 Post hoc analysis - power for 100 participants

Post hoc analysis for 100 subjects indicate that an average sized interaction term can be detected with a power $>$ 0.95, an average sized effect of group can be detected with a power of 0.88, and that an average sized effect of condition can be detected with a power$>$ 0.95.

1.2 General health questions

The questions listed in the Figure S1 were asked before each experiment.

1.3 Anatomical data

The body segments of participants, such as whole trunk, upper arm, forearm were measured in accordance with the body Plagenhoef's segmentation model, Figure S2. The segments masses were calculated with the respect to the statistical data presented in Table S1.

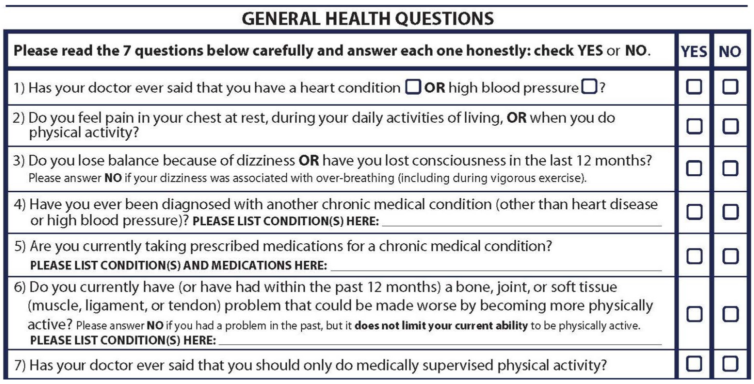

GENERAL HEALTH QUESTIONS

Please read the 7 questions below carefully and answer each one honestly: check YES or NO.	YES	NO
1) Has your doctor ever said that you have a heart condition ☐ **OR** high blood pressure ☐?	☐	☐
2) Do you feel pain in your chest at rest, during your daily activities of living, **OR** when you do physical activity?	☐	☐
3) Do you lose balance because of dizziness **OR** have you lost consciousness in the last 12 months? Please answer **NO** if your dizziness was associated with over-breathing (including during vigorous exercise).	☐	☐
4) Have you ever been diagnosed with another chronic medical condition (other than heart disease or high blood pressure)? **PLEASE LIST CONDITION(S) HERE:** ______	☐	☐
5) Are you currently taking prescribed medications for a chronic medical condition? **PLEASE LIST CONDITION(S) AND MEDICATIONS HERE:** ______	☐	☐
6) Do you currently have (or have had within the past 12 months) a bone, joint, or soft tissue (muscle, ligament, or tendon) problem that could be made worse by becoming more physically active? Please answer **NO** if you had a problem in the past, but it **does not limit your current ability** to be physically active. **PLEASE LIST CONDITION(S) HERE:** ______	☐	☐
7) Has your doctor ever said that you should only do medically supervised physical activity?	☐	☐

Figure S1. The 2020 Physical Activity Readiness Questionnaire for Everyone (PAR-Q+) (Warburton et al., 2019)

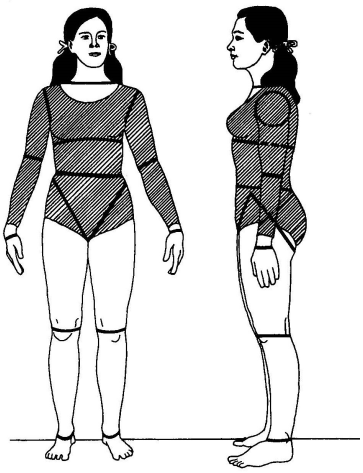

Figure S2. Sixteen body segments (Plagenhoef et al., 1983)

Table S1. Segment masses as percentages of total body mass for males and females (Plagenhoef et al., 1983)

One segment	Men N=35<br>Mean (SD)	Women N=100<br>Mean (SD)
Hand	0.65 (0.06)	0.5 (0.026)
Forearm	1.87 (0.2)	1.57 (0.1)
Upper arm	3.25 (0.49)	2.9 (0.32)
Foot	1.43 (0.13)	1.33 (0.02)
Shank	4.75 (0.53)	5.35 (0.47)
Thigh	10.5 (1.21)	11.75 (1.86)
Whole trunk	55.1 (2.75)	53.2 (4.64)
Head and neck	8.26	8.2
Thorax	20.1	17.2
Abdomen	13.06	12.24
Pelvis	13.66	15.96

1.4 NASA-TLX

Item	Endpoints	Description
Mental demand	1 - 10 Low / High	How much mental and perceptual activity was required (e.g., thinking, deciding, calculating, remembering, looking, searching, etc.)? Was the task easy or demanding, simple or complex, exacting or forgiving?
Physical demand	1 - 10 Low / High	How much physical activity was required (e.g.. pushing, pulling, turning, controlling, activating, etc.)? Was the task easy or demanding, slow or brisk, slack or strenuous, restful or laborious?
Temporal demand	1 - 10 Low / High	How much time pressure did you feel due to the rate or pace at which the tasks occurred? Was the pace slow and leisurely or rapid and frantic?
Performance	1 – 10 Good / Poor	How successful do you think you were in accomplishing the goals of the task set by the experimenter (or yourself)? How satisfied were you with your performance in accomplishing these goals?
Effort	1 - 10 Low / High	How hard did you have to work (mentally and physically) to accomplish your level of performance?
Frustration level	1 - 10 Low / High	How insecure, discouraged, irritated, stressed and annoyed versus secure, gratified, content, relaxed and complacent did you feel during the task?

Figure S3. The description of the subjective demands of the NASA-TLX survey (Hart and Staveland, 1988).

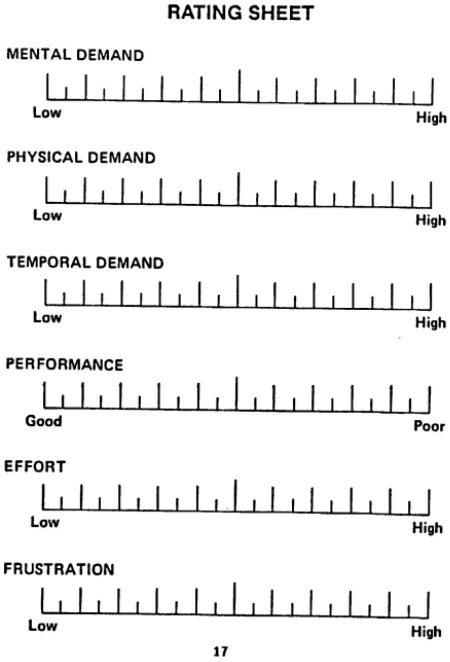

Figure S4. NASA-TLX 100 points rating scale. Questions for rating: Mental demand - How mentally demanding was the task?; Physical demand - How physically demanding was the task?; Temporal Demand - How hurried or rushed was the pace of the task?; Performance - How successful were you in accomplishing what you were asked to do?; Effort- How hard did you have to work to accomplish your level of performance?; Frustration - How insecure discouraged, irritated, stressed, and annoyed were you? (Hart and Staveland, 1988).

We also added information describing the different subjective demands for 6 participants for 3 levels of gravity (1G, 1/3G and 1/6G) for static tasks (S1).

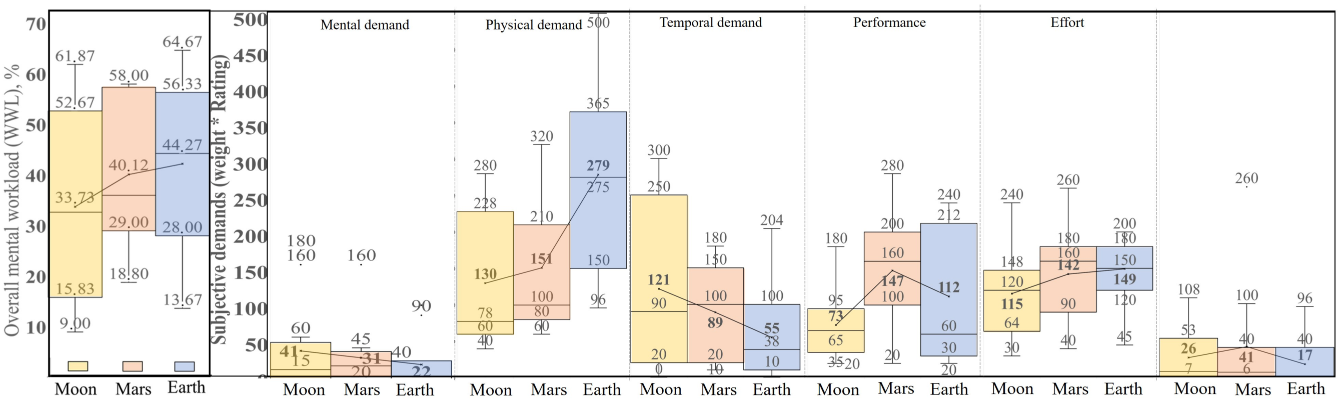

Figure S5. Overall mental workload (WWL,%) and subjective demands components (example for static task (S1)), for males and females for loads (1 kg, 3 kg, 5 kg, 7 kg) - gravity level dependence for static tasks
